# Progress and challenges in the vaccine-based treatment of head and neck cancers

**DOI:** 10.1186/1756-9966-28-69

**Published:** 2009-05-27

**Authors:** Aldo Venuti

**Affiliations:** 1 Laboratory of Virology – Regina Elena Cancer Institute – Via Messi d'Oro 156-00158 Rome, Italy

## Abstract

Head and neck (HN) cancer represents one of the most challenging diseases because the mortality remains high despite advances in early diagnosis and treatment. Although vaccine-based approaches for the treatment of advanced squamous cell carcinoma of the head and neck have achieved limited clinical success, advances in cancer immunology provide a strong foundation and powerful new tools to guide current attempts to develop effective cancer vaccines. This article reviews what has to be rather what has been done in the field for the development of future vaccines in HN tumours.

## Review

The concept that a vaccine could be useful in the treatment of cancer diseases is a long-held hope coming from the observation that patients with cancer who developed bacterial infections experienced remission of their malignancies. In 1896, New York surgeon William Coley locally injected streptococcal broth cultures to induce erysipelas in a patient with an inoperable neck sarcoma, obtaining a tumour regression. Although the therapy was toxic, the patient's tumour ultimately regressed, and he lived disease-free for 8 years before succumbing to his cancer [1]. During the century since Coley's first experiments, immensely more is understood about tumour immunology: the validation of the theory of cancer immunosurveillance, the definition of a large number of tumour antigens as targets for immune recognition, the prognostic significance of immunological parameters, such as the different sub-classes of T cell infiltrating human tumours, and therapeutic benefits of immune-related therapies from BCG to anti-CTLA-4 are the major achievements that pose the theoretical basis to test the validity of cancer vaccines. In particular some characteristics of HNSCC render these tumours susceptibly to explore efficacious immunotherapy: the presence of well characterized Tumour Associated Antigens (TAA) and the possibility to perform clinical trials as adjuvant cancer therapy to eradicate local regional microscopic and micrometastatic disease with minimal toxicity to surrounding normal cells.

## TAA

HNSCC cells, as in general tumour cells, express both unique and shared antigens capable of being recognized by T cells. Identification of CTL epitopes presented by major histocompatibility complex (MHC) class I molecules on tumour cells is vital for the design of active immunotherapy. Many antigens have been identified so far by utilising well characterized approaches already utilised for other tumours. These approaches are:

• A peptide-elution approach involving the biochemical elution of peptides from the binding cleft of tumour HLA molecules, and pulsing these peptides onto APC to test their ability to sensitize target cells for lysis by specific antitumour lymphocytes.

• A reverse immunology approach predicting possible antigenic peptide sequences from oncogenes or tumour-associate proteins using known HLA-anchor motifs, followed by an *in vitro *investigation of the ability of the predicted synthetic peptides to stimulate T lymphocytes.

• A serological approach involving the identification of antigens by recombinant expression cloning (SEREX) [2]. SEREX was developed to combine serological analysis with antigen cloning techniques to identify human tumour antigens eliciting autologous high-titer immunoglobulin G (IgG) antibody responses.

• A genetic approach involving two different methods: i) the transfection of cDNA libraries from tumour cells into target cells expressing the appropriate human leukocyte antigen (HLA) molecule, and then screening transfected cells for stimulating CD8+ T-cell clones from cancer patients; ii) the microarray analyses facilitating the individuation of differential highly expressed genes in HN primary tumour samples [3].

The TAAs that have been described in HNSCC cells are derived from a broad spectrum of intracellular proteins and have bee exhaustively reported in other reviews [3-5]. In principle a complete arrays of TAA antigens can be obtained by immunizing with a heterogeneous mixture of tumour antigens, using irradiated tumour cells themselves or tumour-derived materials such as tumour cell lysates or apoptotic (killed) tumour cells as substrates for generating antitumour immune responses. This approach failed to be effective for many reasons and, mostly, for the clear hurdle represented by the reliance on the proper internalization, processing and antigen presentation by immune cells in which these machineries are already altered in tumour-bearing patients.

In a single patient a particular TAA, not broadly shared among other HNSCC patients, may be detected but the procedures are so laborious to render this approach impractical in clinical application of vaccines.

Significant advances in molecular genetic technology are facilitating the identification of numerous TSAs in head and neck cancer, which try to meet some criteria of an ideal TAA. The ideal TAA must have an unique expression within the tumour or a differential expression as compared to normal tissue or vital organs; it has to be expressed by a majority of head and neck cancers; it should be constitutively expressed and maintained during carcinogenesis; and finally it should be highly immunogenic. To date TAAs matching almost all of these criteria are the human papillomavirus (HPV) E6 and E7 proteins. The association of HPV with HNSCC and the utilisation of viral oncoprotein for immunotherapy has been reviewed elsewhere [6].

Briefly HPV is associated with approximately 20–25% of all HNSCC and up to 60–70% of those tumours localized to the oropharynx, in particular tonsil [7]; the HPV type 16 has been found in more than 90% of HPV-positive HNSCC; the E6 and E7 proteins are constitutively expressed and maintained during the HPV-associated carcinogenesis; and the viral oncoproteins are foreign antigens and, therefore, are highly immunogenic.

Beside the matching to an ideal TAA the HPV E6 and E7 proteins serve as model antigens for the development of immunotherapy and since HPV type 16 is also associated with cervical and anogenital cancers, the same vaccine strategies developed to prevent (already in clinical use) and/or to treat HPV-associated cervical and anogenital cancers can also be used in head and neck cancers [for review see [6,8]]. Nevertheless these oncoproteins account for only 20% of HNSCC and enforces must be done to identify other TAAs in the remaining HNSCC matching closely all the above mentioned criteria. In this filed an enormous work has been done but before some of these TAAs becomes valid therapeutic vaccine other hurdles must be overcome, the tumour immune escape and tumour tolerance.

## Tumour immune escape and tolerance

The discovery of so powerful TAAs in HNSCC is giving substantial basis for efficacious and less toxic treatments, but in the mean time HNSCC as other tumours participates in tumour immune escape through various mechanisms:

i) it disrupts antigen processing and presentation machinery by altering the MHC class I and TAP 1–2 expression;

ii) it recruits immunosuppressive Treg to dampen effector T-cell activity,

iii) by chemokine production it alters T-cell homeostasis increasing the sensitivity of effector T cells to apoptosis.

Downregulation of antigen-processing machinery (APM) components, such as TAP 1/2 and MHC class I antigens, renders ineffective the recognition by CTL in HNSCC. More than 50% of primary and metastatic lesions showed MHC class I antigen loss [9]. Interestingly, interferon-γ (IFN-γ), which functions to up-regulate APM and MHC molecules, can restore *in vitro *the ability of specific CTLs to recognize their tumour cell targets and subsequently to lyse them [10,11]. Thus in a therapeutic setting clinical efforts must be undertaken in order to restore APM and MHC class I antigen expression in HNSCC.

The complex biology of CD4+CD25+FoxP3+ regulatory T cells (Treg), which function to downmodulate immune responses and have enormous implications on the development of cancer immunotherapies, is far to be fully understood.

Tumour cells are believed to recruit Treg within the tumour microenvironment to suppress antitumour immunity. Analyses of tumour-infiltrating lymphocytes revealed a greater percentage of Treg in HNSCC compared with the circulating counterpart of both patient and healthy controls [12], suggesting that in HNSCC Treg cells are recruited in the tumour area respect to the lymphnode or circulating location. Recently, it has been reported that naïve antigen-specific T cells can be either activated or tolerized simultaneously in the same host, depending on the microenvironment in which the epitope is presented [13]. Effector T cells generated in lymph nodes are tolerized rapidly when they infiltrate antigen-expressing tumour tissues. Interestingly, tolerant T cells persist only in the tumours and resemble tumour infiltrating lymphocytes seen in cancer patients [14]. In the clinical setting the effect of Treg may be attenuated by depleting them with non-myeloablative chemotherapy or monoclonal antibodies against inhibitory receptors (anti-CTL antigen-4 [CTLA4]) [15,16]. In various mouse models antibodies against the glucocorticoid-induced tumour necrosis factor receptor family (GITR) are able to downregulate Treg functions increasing the efficacy of immunotherapies [17,18]

However the role of the human counterpart of this receptor huGITR appears to be quite different with less activity on Treg suppression [19,20]

Controlled and effective modulation of Treg cell function for cancer therapeutics will be contingent on a better understanding of the molecular basis of Treg cell interaction with tumour cells and ensuing immunosuppressive mechanisms. A study using a synthetic monoclonal antibody targeted against CD28 met with disastrous results, reminding us that manipulation of costimulatory/regulatory pathways requires more information in this field [21].

Nevertheless continuing investigation on the biology of Treg in antitumour immunity and potential toxicities of Treg suppression will undoubtedly implement the efficacy of cancer immunotherapies.

Finally in patients with HNSCC the absolute number of T-lymphocytes both CD4+ and CD8+ is reduced and it may be related with a decrease expression of chemokine receptor 7 (CCR7) on T cells [22]. CCR7 has been implicated in protecting CD8+ T cells from apoptotic cell death. Indeed CD8+ CCR7-negative T lymphocytes that are more sensitive to apoptosis were increased in HNSCC patient peripheral blood compared with healthy controls [22].

These are the major barriers that have to be broken by an effective therapeutic vaccine. Before reaching the tolerance or tumour escape a therapeutic vaccine must elicit a strong cellular immune response involving the CD4 and CD8 stimulation.

Many strategies have been developed to induce a response against the TAA. In particular the HPV E7 antigen has been utilised to develop an incredible large number of different possible therapeutic vaccines extensively reviewed elsewhere [6]. The basic strategy to induce a specific anti-TAA response has been applied also for the non-viral HNSCC TAA and are briefly discussed.

## Peptide/protein based vaccines

To date, several peptide-based vaccines are either undergoing clinical evaluation or are in development. A major limitation to peptide-based vaccines is the need to identify the immunogenic epitope of the tumour-associated antigen. The observation that the antigenic epitope with the highest binding affinity to the HLA molecule does not necessarily correlate with its potential immunogenicity *in vivo *decreases the applicability of these peptide based vaccines. Thus, MHC molecules may restrict the candidacy for this approach, making difficult to carry out large scale vaccination treatment schemes. The HLA restriction associated with peptide-based vaccines can be overcome with the use of whole protein-based vaccines, harbouring multiple immunogenic epitopes which can bind the various allelic HLA molecules. However, due to the poor immunogenicity of both peptides and proteins most of the researches in this area have focused on the co-administration of adjuvant immune-enhancing agents such as chemokines, cytokines, and costimulatory molecules to enhance the potency of the vaccine [for a review, see [3,23]]. Chimeric GM-CSF molecules can enhance antigenic immune responses through the recruitment of antigen present cells [24,25]; co-administration of immunostimulatory CpG oligodeoxynucleotides may be able to stimulate macrophages to secrete IL-12 shifting the cytokine profiles to a Th1-type cell-mediated immune response [26,27]. Recently the fusion of the beta-1,3–1,4-glucanase (LicKM) of Clostridium thermocellum bacterial protein to the HPV E7 protein produced an antigen with strong intrinsic adjuvating activity, indicating that manipulation of the antigen may elicit some unknown helpful function [28,29]

The results of clinical trials indicate that peptide/protein vaccination has low toxicity but a strong discordance exists between immune and clinical responses, reinforcing the need of further improvement to the vaccination by the utilization of peptide-pulsed dendritic cells, the addition of helper peptides, and depletion of Treg. Several phase I clinical trials using antigenic peptides derived from HPV E6/E7 have been so far conducted as well as multivalent peptide-based vaccination against p53 [30-32] with only "promising" vaccine-induced immunologic responses.

## DNA/RNA based

DNA vaccines have been used in the clinical arena to elicit antigen-specific immune responses. Although nucleic acid vaccines do not appear to induce as vigorous immune responses as live viral vaccine vectors, they have several advantages. Naked DNA is relatively safe, stable, cost efficient, and able to sustain reasonable levels of antigen expression within cells [for review see [33,34]]

DNA-based plasmid vectors remain stable in a wide range of conditions over great lengths of time, and they can be delivered with little risk to individuals who are immunosuppressed. In addition, since DNA vaccines do not elicit neutralizing antibodies in the vaccinated patient, they can be repeatedly administered with similar efficacy.

The cellular machinery is needed to generate tumour antigens and other necessary proteins are provided by the host and not required to be incorporated into the vaccine itself. Finally, the DNA backbone of the injected plasmid contains its own cognate immunostimulatory sequences, which have been shown to activate innate responses [35]. However, disadvantages to DNA vaccines are their relatively low transfection efficiency and poor immunogenicity. Many strategies have been employed to overcome these obstacles mostly trying to produce: an efficient delivery of targeted antigen to antigen presenting cells such as DCs; an enhancement of antigen processing and presentation in DCs; and an augmentation of DC and T cell interaction [36]. Recently, it has been reported that the fusion of the E7 gene of HPV 16 with a plant virus coat protein produced strong antitumour activity in a mouse model activating both CD4+ and CD8+ T cells [37].

A clinical trial with the administration of liposome-encapsulated plasmid IL-2 in combination with chemotherapeutics, was conducted and robust IFN-γ and IL-12 titers were detected in patients with advanced HNSCC [38].

Similarly, phase I clinical trial using a naked DNA vaccine encoding the HPV-16 E7 gene linked to M. tuberculosis HSP70 (pNGVL4a-Sig/E7(detox)/HSP70) is conducting at the Johns Hopkins Hospital (USA) in patients with advanced HPV-16 associated HNSCC. The DNA vaccine was well tolerated and a subset of the vaccinated patients demonstrated detectable systemic levels of E7-specific CD8+ T cell immune responses (M. Gillison and T.C. Wu, personal communication).

## Bacterial/viral vectors

Bacteria, such as Listeria monocytogenes, Salmonella, Lactococcus lactis, Lactobacillus plantarum, Bacillus Calmette-Guerin, and several viral vectors, including vaccinia virus (VV), adenovirus, adeno-associated virus, alphavirus, and its derivative vectors, such as sindbis virus, semliki forest virus, and venezuelan equine encephalitis virus have been used to deliver genes or proteins of interest to elicit antigen-specific immunotherapy [for review, [39]].

Among the bacterial vectors, L. monocytogenes has emerged as a promising vector, because in animal models it is able to induce both CD8+ and CD4+ immune responses to elicited regression of established tumours, and to overcome central tolerance by expanding low avidity CD8+ T cells specific for E7 [40].

Among viral vectors, VV was historically one of the first viral vector employed in clinical trials of therapeutic vaccines against HPV-associated cancer [41]. To date many VV vaccines have been employed in clinical trials to deliver genes and antigens of interest efficiently. VV was utilised to express HPV-16/18 E6/E7 fusion protein, termed TA-HPV, that was able to induce T cell mediated immune responses [42-46] or to express HPV E2 protein, called MVA-E2, that generated a HPV specific cytotoxic response against the papilloma-transformed cells which resulted in regression of high-grade lesions [47-49]. The progression of the genital tumour clinical trials using these bacterial/viral vectors encoding HPV antigens will elucidate the possible applicability to the HPV-related subset of HN cancers.

## Plant-derived/produced antigens

Since ancient times plants have been used for therapeutic purposes, mostly by providing medicinal compounds that have been extracted and used to treat illness. Nowadays, plant molecular farming provides new therapeutic possibilities combining the innovations in medical science and plant biology to create affordable pharmaceutical products. Many methods are available for the antigen production and all the TAA antigen in principle can be obtained with the available technologies [50].

The simple demands for solar light, water and minerals make plants an easier and more economical system for the production of heterologous proteins than industrial facilities using fermentation technology. It is estimated that recombinant proteins can be produced in plants at 2–10% of the cost of microbial fermentation systems and at 0.1% of the cost of mammalian cell cultures. Yields of 0.1–1.0% total soluble protein are sufficiently competitive with other expression systems to make recombinant plants economically viable [51]. Moreover, scale-up technology is available for harvesting and processing plants or plant products on a large (potentially agricultural) scale. Beside the cost-effectiveness of plant production, plant derived antigens seem to possess intrinsic activity that may enhance their immunogenecity. A tumour idiotype-specific scFv epitope from a mouse B cell lymphoma, that was produced at high levels in tobacco plants *(N. benthamiana) *and utilized as therapeutic lymphoma vaccine in subcutaneous immunization, induced an anti-idiotype immune response and protected mice from challenge by a lethal dose of syngeneic tumour cells. Interestingly, mice that received the scFv alone, without adjuvant, showed a high degree of protection [52], indicating that either the proper conformation or some other unknown factor provided by the plant-expression system, improved the efficacy of the immunogen. The same adjuvant-like effect was noticed when other plant-produced human scFvs (cloned from tumour biopsy cells), purified from the interstitial fraction were tested in mice for appropriate anti-idiotype response [53]. These plant-produced scFvs are currently undergoing phase I clinical trials. A colorectal cancer antibody [54] and a colorectal cancer antigen [55] have been also produced in *N. benthamiana *by a TMV-based vector. The purified plant-derived tumour antigen was able to stimulate T cells and indicated the presence of some adjuvant-like effect. Recent data indicate that adjuvant-like effects were obtained in immunizations with crude plant extract containing the E7 protein of HPV16. The recombinant plant-derived vaccines as '*in planta *formulation' without adjuvants were able to elicit also a protective Th1 cell response in mice [56]. Same adjuvanting activity was seen with another plant-produced fusion protein of the HPV16 E7; this antigen preparation was able to induce a specific CD8+ T stimulation that elicit a therapeutic affect on experimental tumours [28].

These promising results in pre-clinical models are the basis to undertake phase I-II clinical trials in HNSCC.

## Dendritic cell based

Among specialized APCs the most potent are DCs because they express high levels of MHC and costimulatory molecules. Therefore on DCs were focused the research of many investigators and a variety of methods for generating DCs, loading them with tumour antigens, and administering them to patients were developed. In fact, in murine models of HNSCC DCs, pulsed with apoptotic tumour cells and activated with interleukin-2, induced strong antigen-specific anti-tumour immunity [57].

*Ex vivo *loading of DCs may be achieved by proteins or peptides, or tumour cells, or genomic DNA transfection, or genetically engineered vectors, or cell fusion techniques. By these methods a pool of uniform, controlled, and optimally activated APCs can be generated, suggesting a positive utilisation as therapeutic vaccines. Nevertheless the requirement of expensive GMP facilities have discouraged clinical investigators to implement phase I trials.

Recent studies have shown that DC therapy produces the regression of both established carcinomas and haematologic malignancies [58,59].

At least three examples of DC vaccine therapy in HNSCC have been reported [5]. In the first attempt the DCs were pulsed with autologous tumour cells but the trial was interrupted because was quite impossible to obtain 10^7 ^tumour cells in sterile conditions for vaccination and the DTH evaluation of the patients suggested that this strategy is an unlikely candidate for large scale application. The second attempt with DCs electroporated with genomic DNA from autologous tumour cells overcame this problem and a Phase I trial is in progress. In the third attempt the DCs were loaded with sequence of wild type (wt) p53 peptides on the basis that the majority of HNSCCC over express this oncoprotein and clinical trials are underway. For the subset of HPV-related HN cancers DCs, pulsed with recombinant HPV-16 and HPV-18 E7 proteins, have been evaluated in patients with advanced HPV-associated anogenital cancers [60]. In general, the vaccine was well tolerated with no significant local or systemic side effects and HPV antigen-specific T cell responses were observed in some of the patients [61].

Data from this early stage of clinical development indicate that a fraction of patients, often less than half, exhibits immune responses against the vaccinating antigen, indicating that DC vaccines, as other vaccination strategies, require further improvement, perhaps by exploring the most effective route of administration, vaccination schedule, prime-boost regimens, and various maturation protocols.

## Tumour-cell based vaccines

Although immunization using autologous irradiated tumour cells can deliver a range of tumour antigens to the immune system that may not be present in single-target vaccines and is avoiding the challenges involved in *ex vivo *propagation of tumour or immune cells, the poor expression, processing and presentation of TAA by tumour cell itself leads to ineffective immunization. Consequently, studies have focused on strategies to enhance the potency of cell based vaccines including transduction of tumour cells with MHC or costimulatory molecules, co-administration of adjuvants such as Bacillus Calmette-Guerin, and engineering tumour cell vaccines to secrete immunostimulatory cytokines.

Among the immunostimulatory cytokines that have been employed in transducing tumour cells, the GM-CSF showed the most promising results [for review, [61]]. GM-CSF can be also produced by mixing irradiated tumour cells with controlled GM-CSF releasing microspheres or bystander GM-CSF producing cells. Tumour cells have been also engineered to express MHC and/or co-stimulatory molecules, such as B7-1 [62,63] in order to activate immune cells. None of these techniques have been applied so far to HN cancer, nevertheless tumour-cell based vaccines represent an attractive approach which merits further investigation in order to overcome the hurdle represented by the need to obtain tumour tissue from each patient.

## Adoptive transfer of active T cells

All the above mentioned vaccine preparation can reach a strong CTL stimulation in vaccinated animals or humans. However, even high levels of CTL did not correlate with the presence of active effector cells within the tumours as the tumour escaping mechanisms are actively fighting the CTL induced by the TAA utilised for immunotherapy. The adoptive transfer of active T cells may overcome the immunotolerance obstacle. This technique relies on the *ex vivo *activation and expansion of tumour-reactive lymphocytes which are then returned to the host.

Poorly immunogenic established tumours have been cured by ACT in murine models [64-66]. Consequently, similar strategies were transferred into the clinical setting but early studies demonstrated only partial success [67-71]. In more recent approaches ACT was utilised together with strategies to deplete the immune system of endogenous T-cell subpopulations like naturally occurring T regulatory cells or to limit the physical space in transferring cells [71,72]. By these approaches first successful therapy was reported in a single patient with melanoma metastasis [73] and thereafter in 35 patients was demonstrated an objective clinical response in over 50% of them [74,75].

Although these studies, for the first time in humans, demonstrate that ACT is a viable therapeutic strategy, it has to be pointed out that results were achieved in the setting of a combined approach with chemotherapy as immunomodulatory agent, indicating the pivotal role of modulating T-reg and suggesting that in this setting even other vaccine therapy could be efficacious. Furthermore for the treatment or prevention of HNSCC it is important to note that ATC as well as DC strategies require cellular products that are subject to individual patient variability, and the differences in culture methods, loading strategies, and injection techniques render these approaches hard to be transferred to phase II/III studies and posing formidable challenges to large-scale clinical implementation.

## Antibodies against functional molecules of the tumour

Targeting HNSCC cell surfaces with high-affinity antibodies is a total different approach that is emerging as advantageous strategy in the development of immunotherapies. mAb therapy is based on multiple mechanisms of action including: inhibition of ligand induced activation; induction of receptor degradation or complement-mediated/antibody-dependent cellular cytotoxicity; activation of tumour-specific CTL via cross-priming of lysed tumour cells; and finally delivering of a conjugated chemotherapeutic toxin to the tumour bed when linked to the antibody [76-78].

To date, most of the mAb therapies target the EGFR as this receptor is overexpressed in more than 90% of HNSCC [for review, [6,79]].

Cetuximab, a chimeric IgG1 isotype murine/human epidermal growth factor receptor-specific monoclonal antibody, as well as has Panitumumab, a fully humanized IgG2 isotype monoclonal antibody, have been approved by the US Food and Drug Administration, and their clinical efficacy is well documented [80]. It is possible that these monoclonal antibodies, employed to block the signalling pathways, may also serve as immunostimulants. The Fc portion of monoclonal antibodies binds to the Fcγ receptor (FcγR) of effector cells like natural killer cells, macrophages/monocytes, and other granulocytes, recruiting these cells that participate in antibody-dependent cellular cytotoxicity by the release of lytic mediators for the target cells. Indeed, polymorphisms in the Fcγ receptor can predict clinical outcomes in patients with metastatic colorectal cancer receiving cetuximab therapy [81].

Antibodies that may have an immunostimulatory component have been developed against another overexpressed tumour antigen, the vascular endothelial growth factor (VEGF) which is a tumour secreted molecule that stimulates angiogenesis and lymphangiogenesis. High expression of VEGF and its receptor was detected and associated with poor survival in patients with head and neck cancers [82]. Bevacizumab is a recombinant humanized anti-VEGF mAb which is currently being evaluated in several tumours with promising results but only in term of trends [for review, [81]]. This therapy has yet to be explored in head and neck cancers.

Finally antibodies can be targeted to molecules involved in immune modulation. In mice antitumour immunity was achieved by antibodies against the CTLA4 molecule that is a ligand expressed by T lymphocytes that functions to inhibit T-cell activation by binding to B7.1 and B7.2, thereby preventing CD28 from binding to B7 [83].

The brilliant results of a phase 1 clinical trial using a fully humanized antagonistic CTLA4 monoclonal antibody highlight the potential immunotherapeutic value of antibody-based therapies for cancer [16].

## Future challenges and progresses

The introduction in the clinical practice of two highly efficacious preventive vaccines [84,85] (Gardasil MSD, and Cervaix GSK) against HPV opens a new scenario suggesting a role of this vaccination in the preventive therapy of the subset of HNSCC linked to HPV infection, hypothesising a preventive immunological approach for other tumours. Trials to evaluate prevention require greater numbers of participants, longer follow-up to evaluate meaningful endpoints, and raise different ethical issues than therapeutic studies. However it is predictable that not all tumours can beneficiate of this preventive approach, stressing the need for cancer immunotherapies.

Cancer vaccines are a powerful example how is wrong to approach to scientific problems by optimism or pessimism about the initial results. The degree of optimism or pessimism associated with researches into therapeutic cancer vaccines depends largely upon definitions of response to treatment. If you use objective complete response and partial response to cancer vaccines as indicated by World Health Organization (WHO) [86] the pessimism is compulsory; if you consider the Response Evaluation Criteria in Solid Tumours (RECIST) [87] cautious optimism or less pessimism is conceivable, whereas if less objective so-called "soft" criteria are employed (e.g. minor response, stable disease, clinical benefit) are employed the optimism about immunotherapy predominates.

Data of phase I-II trials with these large arrays of therapeutic vaccines indicate their efficacy in elicit some immunological response, and only few phase III trials reported success in the therapy having the RECIST as end point.

In a recent reviews for all type of tumours a percentage of only 2.9% of clinical response to therapeutic vaccines was reported [88,89]. However, results from cancer immunotherapy must be viewed in the context of the patient populations included in trials. Indeed, response rates will be low if the enrolled patients have metastatic disease with failure after standard therapies [90].

Therefore the pessimistic and simply conclusion that cancer vaccines have been tested and failed may be wrong. Only in relative short time the knowledge on immunotolerance and tools to overcome it have been achieved, emphasizing the need for profound changes in the application of immunotherapy.

Firstly, investigators have to concentrate their efforts in:

• Generating antitumour CD4+ cells that enhance antitumour reactions and sustain the activation and survival of CD8+ cells.

• Activating innate immunity by new toll-like [91] receptor agonists.

• Targeting TAA that are essential for tumour cell survival.

• Stimulating inflammatory response/environment at the tumour site.

• Utilising adjuvated antigens to activate quiescent cells (e.g. with costimulatory molecules).

• Blocking negative costimulatory molecule (the already reported efficacy of anti CTLA-4 monoclonal antibodies holds promise for this approach [for review, [92]).

• Finally eliminating both tumour and Treg-mediated immune suppressive mechanisms without adversely affecting effector cells, that recent evidence indicates as the most importantly achievement [93].

Secondly, wide-scale evaluation and clinical application of cellular-based vaccines are limited by factors such as product uniformity and the significant resources necessary for successful production. Efforts must be done in order to overcome the technology obstacles limiting the development of T-cell based vaccines as standardized reagents. Moreover even the other immunotherapeutic approaches need the development of standardized procedures and vaccines to be evaluated in multi-institutional studies. The future success of immunotherapy will depend mostly on standardization.

Thirdly, when used in the therapeutic setting, it is now clear that antitumour immunity can be augmented by ancillary approaches such as prime-boost strategies, or multivalent vaccines, or the use of chemotherapeutics or molecules which regulate costimulatory functions or different route of delivery. The last issue may hold promise as a mean of enhancing vaccine efficacy. Classical antimicrobial vaccination strategies have relied on subcutaneous or intramuscular injections to stimulate long-lasting immunity. However, it is now clear that the route of vaccination impacts both the potency and location of immune response generated. DNA immunization elicits completely different response if the same antigen encoding plasmid is injected intradermally, subcoutaneously or intramuscularly. In mouse model, subcutaneous injection of DC causes the T-cell responses and the localization of DC into the draining lymph nodes whereas intravenous administration does not [94]. Intratumoural boosting shots produce better antigen-specific T-cell responses [95]. In the clinical setting, various studies indicated that DNA vaccines [96], DC [97], or autologous tumour cells [98] delivered by intranodal and intralymphatic injections yielded improved CTL responses in cancer patients. Oral administration is another fascinating hypothesis of tumour vaccination as well as the utilisation of edible vaccines and, in this issue, some evidence is coming out [50]. However, only a small number of studies have correlated vaccination route with memory T-cell function and therefore efforts must be done in introducing this variable in the experimental setting

## Conclusion

While immune therapy for the treatment of cancer holds promise, current cancer vaccines have broad limitations and few objective clinical responses.

Nevertheless immunotherapy can be successful in cancer patients and thus increased effort in the development of cancer immunotherapy is needed. Further clinical studies should utilize standard criteria for clinical response and require validation in increased numbers of patients.

Now, where are we? We have climbed the K2 mountain (the individuation of useful TAA and of vaccine settings able to induce CTL response) and we are climbing the Everest mountain (the tumour immunotolerance and immune escape).

## Abbreviations

ACT: adoptive cell transfer; APC: antigen-presenting cell; APM: antigen-processing machinery; BCG: Bacillus Calmette Guerin; CCR7: chemokine receptor 7; CTL: Cytotoxic T cell; CTLA4: anti-CTL antigen-4; DC: dendritic cell: GITR: glucocorticoid-induced tumour necrosis factor receptor family; GM-CSF: Granulocyte-macrophage colony-stimulating factor; GMP: good manufacturing practice; HLA: human leukocyte antigen; MHC: major histocompatibilty complex; HN: head neck; HNSCC: head neck squamous cell carcinoma; HPV: human papillomavirus; HSP70: heat shock protein 70; IFN: interferon; RECIST: Response Evaluation Criteria in Solid Tumours; SEREX: serological analysis of recombinant cDNA expression; TAA: tumour associated antigen; TAP: transporters associated with antigen processing; TSA: tumour-specific antigen; TMV: tobacco mosaic virus; Treg: regulatory T cells; VEGF: vascular endothelial growth factor; VV: vaccinia virus; WHO: World Health Organization.

## Competing interests

The author declares that they have no competing interests.
